# Changes of Some Hormones Levels in Patients With Hepatitis B Virus-Related Chronic Liver Disease

**DOI:** 10.4021/gr532w

**Published:** 2013-09-09

**Authors:** Ayfer Serin, Mesut Akarsu, Hale Akpinar, Ilkay Simsek

**Affiliations:** a Departmet of Gastroenetrology, Ege University, Bornova, Izmir, Turkey; b Department of Gastroenterology, Doluz Eylul University, Inciralti, Izmir, Turkey

**Keywords:** Hepatitis B, Hormones, Chronic liver diseases

## Abstract

**Background:**

The purpose of the study was to evaluate some of the hormones in patients with chronic liver disease and cirrhosis.

**Methods:**

The men patients with chronic hepatitis B (Group 1), liver cirrhosis secondary to hepatitis B (Group 2), were included in this study. Additionally, a control group of healthy volunteers (Group 3) was formed. We investigated serum levels of Follicle-stimulating hormone (FSH), Luteinizing hormone (LH), Total testosterone (T. TES), Free-testosterone (F. TES), Estradiol (E2), Androstenedione (ANDR), Dihydroepiandrosterone (DHEA), Progesterone (PPOGES), Prolactin (PRL), Sex hormone binding protein (SHGB) were measured by radioimmunoassay and chemiluminescent immunoassay methods.

**Result:**

A total of 73 patients with chronic liver disease and cirrhosis were included in the study. Patients were grouped as cirrhosis (n = 28), chronic hepatitis B (n = 41) according to the type of their chronic liver disease. Serum F.TEST level in patient groups (group 1, group 2) was found to be lower than control group (P = 0.045, P = 0.047). Serum LH value was found to be higher in patient group (group 2) than control group (P = 0.048). Serum estradiol was higher in the group 2 compared to the control group (P = 0.046).

**Conclusions:**

The described disturbances of some of the observed hormones (LH, E2, F. TES) are complex, particularly in their relationship by which the clinical picture of the hepatitis B related cirrhotic patients and chronic liver disease can be explained.

## Introduction

Hepatitis B virus (HBV) infection is a serious public health problem affecting more than 400 million people worldwide and is at risk of developing liver cirrhosis and hepatocellular carcinoma. Each year more than one million people die from HBV-related liver diseases [1-4]. Trials have been performed on changes in gonadal hormones in chronic liver disease and cirrhosis. Most of these trials refer to alcohol-related chronic liver diseases and cirrhosis. There are only a few similar studies for patients with chronic liver disease and cirrhosis associated with viral hepatitis [4].

It is known that testosterone biosynthesis and secretion in testes are reduced, abnormalities in Leydig cell morphology are demonstrated and germ cell loss in seminiferous tubules is observed in patients with alcohol-related chronic liver disease and cirrhosis. As a result of these changes, testicular atrophy and decreased testosterone levels are observed. Feminization occurs due to increased estrogen biosynthesis [5].

Additionally, disturbances in gonadal hormones were detected during the course of other chronic diseases like chronic renal failure - except cirrhosis. It was also demonstrated that stress leaded to a long-term decrease in LH (luteinizing hormone) in males after a transient increase and consequently to a decrease in testosterone levels [6].

In cirrhosis, excess production of SHBG in liver and increased prolactin levels were detected while exploring the cause of gynecomastia and high level of liver estrogen receptors was added to the direct suppressing effect of estrogen on Leydig cell functions [7].

Findings of testicular atrophy and feminization were observed in patients with advanced cirrhosis. In histological studies exploring testicular tissues of patients with hemochromatosis, atrophy of seminiferous tubules and absence of spermatozoa and spermatid were demonstrated, while similar findings were observed in patients with alcohol-related liver cirrhosis [8, 9].

Positivity rate for liver estrogen receptors was found to be high in patients with chronic hepatitis B and C, with the highest rate found in the patients with HCC [9]. High estrogen levels were also detected in patients with alcohol-related liver cirrhosis [10]. Additionally, hyperestrogenism was observed in patients with idiopathic hemochromatosis and non-alcoholic liver cirrhosis.

The purpose of the study was to evaluate some of the hormones in patients with chronic liver disease and cirrhosis.

## Materials and Methods

Among the patients admitted into Dokuz Eylul University School of Medicine, Department of Internal Medicine, Department of Gastroenterology, Hepatology Clinic between 2003 and 2006, the men patients ranging from 19 to 65 years in age with chronic hepatitis B (Group 2), liver cirrhosis secondary to hepatitis B (Group 3), were included in this study. Additionally, a control group of healthy volunteers (Group 4) was formed. After obtaining informed consent forms from these patients, their histories were collected and physical examinations were performed. All biochemical, microbiological, radiological and pathological (liver biopsy results) data on these patients’ files were reviewed. After evaluation of the patients with data on their files, laboratory results and physical examinations, they were grouped with the related diagnosis of Child A, B and C cirrhosis.

The antiviral and interferon treatments received by these patients were found out from their files. Serum levels of Follicle-stimulating hormone (FSH), Luteinizing hormone (LH), Total testosterone (T. TES), Free testosterone (F. TES), Estradiol (E2), Androstenedione (ANDR), Dihydroepiandrosterone (DHEA), Progesterone (PPOGES), Prolactin(PRL), Sex hormone binding protein (SHGB), Free triiodothyronine (FT3), Free thyroxine (FT4), Thyroid stimulating hormone (TSH), α-fetoprotein (AFP) were measured by radioimmunoassay and chemiluminescent immunoassay methods after collecting venous blood from the patients’ peripheral vessels. The study was generated as a retrospective study.

### Inclusion criteria

The study was intended to be performed in a hospital setting with a control group. Patients were required to give their written informed consent forms. Patients with Child-Pugh A, B and C cirrhosis related to hepatitis B, and chronic hepatitis B, hepatitis B carriers were included in the study.

### Exclusion criteria

If patients were considered to have a primary diagnosis of another endocrinological disease, or other medical, physiological and social problems preventing inclusion, or results of physical examination and laboratory tests during screening hindering the study, then those cases were excluded.

Patients with hepatitis B carriers, chronic hepatitis B, HBV-related liver cirrhosis (Child A, B, C) were defined as group 1, group 2 respectively, where as the control group was defined as group 3. The acquired data was evaluated using Kruskal Wallis test and Mann-Whitney U methods and the relation of each group with the control group was studied the values of P < 0.05 statistically were considered as significant, where as P > 0.05 was insignificant.

## Results

Total 73 patients 45 chronic hepatitis B, 28 patients with HBV-related cirrhosis. Among the patients with HBV-related liver cirrhosis, 13 patients had Child A, 9 patients had Child B and 6 patients had Child C liver cirrhosis, 38 individuals were included in the control group. Patients with a history of treated or under treatment endocrine diseases were excluded in this study.

The median ages for included groups were 52 (26 - 68) years for group1, 57 (34 - 66) years for group 2, 53 (24 - 72) years for group 3, with no difference between the groups with respect to median ages (P ≥ 0.05).

**Figure 1:**
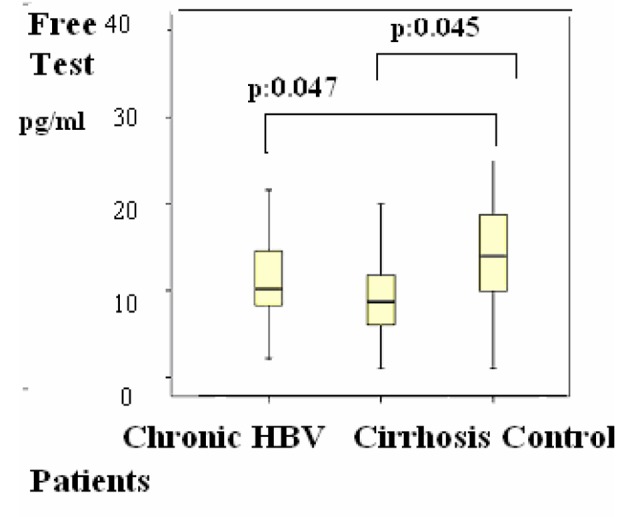
Figure 1. Distributions of Free testosterone (F. test) levels in patients. Differences were assessed using Kruskal-Wallis tests and Mann-Whitney U test.

Group 1 were compared with group 2, the differences of the subjects were shown in Table 1, P < 0.05 values were considered to be statistically significant. According to these results, F. TES was 10 (2 - 20) pg/mL versus, 14 (1 - 34) pg/mL (P = 0.045) (Fig. 1); and these results were statistically significant.

**Table 1:** Distributions of Serum Hormones Levels in Patients

Hormones	Group 1 Median (range)	Group 2 Median (range)	Group 3 Median (range)
FSH (mIU/mL)	4 (1 - 19)	5 (1 - 39)	6 (1 - 31)
LH (mIU/mL)	3 (1 - 7)	4 (1 - 20) *	3 (1 - 8)
T. TES (ng/dL)	496 (255 - 1032)	417 (28 - 1,062)	442 (189 - 846)
F. TES (pg/mL)	8 (1 - 20)	10 (2 - 21)*	14 (1 - 34) *
E2 (pg/mL)	20 (20 - 77)	22 (20 - 47) *	20 (20 - 39)
ANDR (ng/mL)	2 (1 - 7)	2 (1 - 5)	2 (1 - 8)
DHEA (µg/dL)	145 (20 - 492)	52 (15 - 231) *	163 (39 - 528)
PROGES (ng/mL)	0.4 (0.2 - 7)	0.3 (0.2 - 1.3)	0.3 (0.2 - 1.3)
PRL (ng/mL)	6 (1 - 50)	8 (2 - 32)	7 (1 - 39)
SHBG (nmol/mL)	42 (21 - 79)	48 (2 - 114)	41 (15 - 100)

FSH: Follicle-stimulating hormone; LH: Luteinizing hormone; T. TES: Total testosterone; F. TES: Free testosterone; E2:estradiol; ANDR: Androstenedione; DHEA: Dihydroepiandrosterone; PROGES: Progesterone; PRL: Prolactin; SHBG: Sex hormone binding protein. * : P < 0.05 Kruskal-Wallis tests and Mann-Whitney U test.

**Figure 2:**
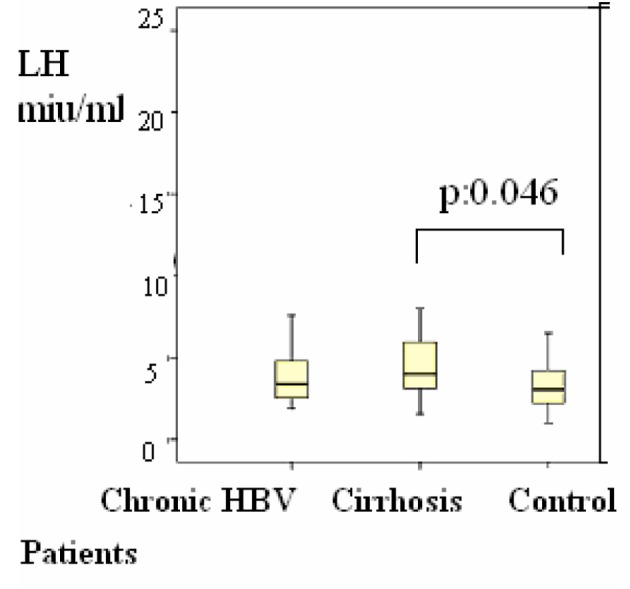
Distributions of serum luteinizing hormone (LH) levels in patients Differences were assessed using Kruskal-Wallis tests and Mann-Whitney U test.

**Figure 3:**
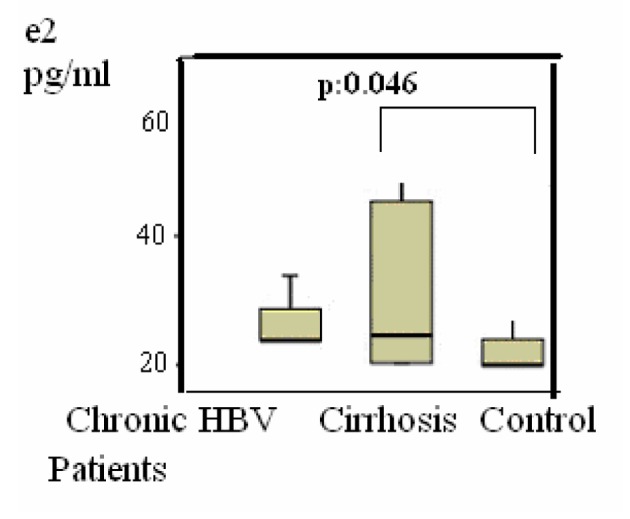
Distributions of Estradiol (E2) levels in patients. Differences were assessed using Kruskal-Wallis tests and Mann-Whitney U test.

Group 2 were compared with group 3, the differences of the subjects were shown in Table 1, P < 0.05 values were considered to be statistically significant. According to these results, LH was 4 (1 - 20) mIU/mL in the group 2 versus group 3 (1 - 8) (P = 0.048) (Fig. 2); estradiol was 21 (20 - 77) pg/mL versus 20 (20 - 39) pg/mL (P = 0.045) (Fig. 3); F. TES was 8 (1 - 20) pg/mL vs. 14 (1 - 34) pg/mL (P = 0.047) (Fig. 1) and these results were statistically significant.

## Discussion

In this study, LH value was found to be higher in the patient group 2 than the control group (group 3).

Serum free testosterone level in patient groups (group 1, group 2) was found to be lower than the control group (group 3).

According to these results, testosterone levels were low but LH level were high in the above-mentioned patient groups. This was similar to the hormonal changes observed in patients with primary hypogonadism. These results were also reported in patients with cirrhosis induced by chronic alcohol use. It was known that testosterone secretion and biosynthesis were reduced, abnormalities in Leydig cell morphology were demonstrated and germ cell loss in seminiferous tubules was observed in these patients. Steenbergen W. noted disorders in endocrine functions of the gonadal system of cirrhotic patients - especially of male and female patients with alcohol-related cirrhosis. The effect of alcohol on primary gonadal insufficiency was associated with the toxic effect of ethanol or acetaldehyde on gonads [11]. FSH, LH and testosterone results obtained in another study included chronic liver disease with causes other than idiopathic hemochromatosis, and primary gonadal insufficiency in cirrhotic patients. There was insufficient response in Leydig cells for external gonadotropin stimulation of these patients [12-14].

Furthermore, Wang YJ et al studied gonadal dysfunction and changes in cirrhotic patients and evaluated sex hormones of cirrhotic patients with liver cirrhosis related to hepatitis B (n = 27) and with alcohol-related liver cirrhosis (n = 21). They added a control group to the study and compared the results of this control group and patient groups. There was no difference with respect to test results between the patient groups; however, significant differences in FSH, LH, prolactin, estradiol and testosterone levels were observed between the patient groups and the control group. Decreased serum testosterone level and increased estradiol and prolactin levels were observed in both groups with alcoholic and HBV-related liver cirrhosis, compared to the control group. Low testosterone and high prolactin levels were determined to be correlated with the severity of cirrhosis. FSH and LH levels were not determined to be high in cirrhotic patients; response was obtained against the external gonadotropins and serum testosterone levels increased [6].

Wang YJ et al carried out a study on patients with HBV-related liver cirrhosis and alcohol-related liver cirrhosis [15]. We obtained results similar to primary hypogonadism in our patient group - similar to the findings obtained in researches on patients with alcoholic liver cirrhosis and with liver cirrhosis related to idiopathic hemochromatosis. However, the study conducted by Wang YJ et al revealed low FSH and LH values, as alcohol had direct toxic effect on the gonads as well as effects on the hypothalamic-pituitary-gonadal axis [15].

According to our results, serum estradiol was higher in the group 2 compared to the control group (group 3). Chopra et al stated that feminization findings in chronic alcohol abusers resulted from high levels of estradiol [15, 16]. Van Thiel et al reported that testicular atrophy and testosterone levels decreased after stopping the portal blood circulation in male rats. They stated that this might lead to peripheral aromatization of androgens, resulting in conversion of testosterone to estradiol [17]. Gonadal dysfunction may develop in chronic alcohol abuse and alcohol-related cirrhosis. It is known that testosterone biosynthesis and secretion are reduced, abnormalities in Leydig cell morphology are demonstrated and germ cell loss in seminiferous tubules is observed in these patients. As a result of these changes, testicular atrophy and decreased testosterone levels are observed. Feminization occurs due to increased estrogen biosynthesis. In addition, ethanol increases estrogen sensitivity in sex hormone-sensitive tissues. Levels of non-receptor cytosolic estrogen-binding protein also decrease due to cirrhosis [18]. Furthermore, F. Farnetti et al studied estrogen receptor expression, estrogen receptor type and oxidative DNA damage in patients with HBV- and HCV-related chronic liver diseases, suggesting that positivity for an estrogen receptor variant in liver might lead to high genomic damage and higher levels of cytoproliferation and carcinogenesis [10]. It was observed that peripheral conversion of androgens to estrogen increased due to high aromatase activity [7, 16] and estrogen levels increased, especially in patients with alcohol-related chronic liver diseases [8]. It was also stated that conversion of adrenal androstenedione to estrone increased in cirrhotic patients [19]. As a consequence of these information and studies, the similar high level of estradiol in our patient group complied with the previous studies.

In conclusion, it was determined by this study that gonadal hormone disorders proven in alcohol-related liver cirrhosis also developed in HBV-related liver diseases.

## Abbreviations

HBV: Hepatitis B virus
FSH: Follicle-stimulating hormone
LH: Luteinizing hormone
T. TES: Total testosterone
F. TES: Free testosterone
E2: Estradiol
ANDR: Androstenedione
DHEA: Dihydroepiandrosterone
PPOGES: Progesterone
PRL: Prolactin
SHGB: Sex hormone binding protein
FT3: Free triiodothyronine
FT4: Free thyroxine
TSH: Thyroid stimulating hormone
AFP: α-fetoprotein

## References

[1] Lee WM (1997). Hepatitis B virus infection. N Engl J Med.

[2] Leemans W, Janssen HL, de Man R (2007). Future prospectives for the management of chronic hepatitis B. World J Gastroenterol.

[3] Lok AS, McMahon BJ (2007). Chronic hepatitis B. Hepatology.

[4] Fattovich G, Bortolotti F, Donato F (2008). Natural history of chronic hepatitis B: special emphasis on disease progression and prognostic factors. J Hepatol.

[5] Vermeulen A (2000). Andropause. Maturitas.

[6] Ferrini M, Wang C, Swerdloff RS, Sinha Hikim AP, Rajfer J, Gonzalez-Cadavid NF (2001). Aging-related increased expression of inducible nitric oxide synthase and cytotoxicity markers in rat hypothalamic regions associated with male reproductive function. Neuroendocrinology.

[7] Terasaki T, Nowlin DM, Pardridge WM (1988). Differential binding of testosterone and estradiol to isoforms of sex hormone-binding globulin: selective alteration of estradiol binding in cirrhosis. J Clin Endocrinol Metab.

[8] Van Thiel DH, Gavaler J, Lester R (1974). Ethanol inhibition of vitamin A metabolism in the testes: possible mechanism for sterility in alcoholics. Science.

[9] Van Thiel DH, Lester R (1974). Editorial: Sex and alcohol. N Engl J Med.

[10] Farinati F, Cardin R, Bortolami M, Grottola A, Manno M, Colantoni A, Villa E (2002). Estrogens receptors and oxidative damage in the liver. Mol Cell Endocrinol.

[11] Kley HK, Niederau C, Stremmel W, Lax R, Strohmeyer G, Kruskemper HL (1985). Conversion of androgens to estrogens in idiopathic hemochromatosis: comparison with alcoholic liver cirrhosis. J Clin Endocrinol Metab.

[12] Van Steenbergen W (1993). [Alcohol, liver cirrhosis and disorders in sex hormone metabolism]. Acta Clin Belg.

[13] Geisthovel W (1979). Hypothalamic-pituitary function (LH, FSH and prolactin) in males with chronic liver diseases. Z Gastroenterol.

[14] Geisthovel W, Perschke B, von zur Muhlen A, Klein H (1979). [Plasma testosterone, free testosterone fraction LH and FSH in males during the early stage of acute myocardial infarction (author's transl)]. Z Kardiol.

[15] Wang YJ, Wu JC, Lee SD, Tsai YT, Lo KJ (1991). Gonadal dysfunction and changes in sex hormones in postnecrotic cirrhotic men: a matched study with alcoholic cirrhotic men. Hepatogastroenterology.

[16] Chopra IJ, Tulchinsky D, Greenway FL (1973). Estrogen-androgen imbalance in hepatic cirrhosis. Studies in 13 male patients. Ann Intern Med.

[17] Van Thiel DH, Lester R, Sherins RJ (1974). Hypogonadism in alcoholic liver disease: evidence for a double defect. Gastroenterology.

[18] Santucci L, Graham TJ, Van Thiel DH (1983). Inhibition of testosterone production by rat Leydig cells with ethanol and acetaldehyde: prevention of ethanol toxicity with 4-methylpyrazole. Alcohol Clin Exp Res.

[19] Nguyen HV, Mollison LC, Taylor TW, Chubb SA, Yeap BB (2006). Chronic hepatitis C infection and sex hormone levels: effect of disease severity and recombinant interferon-alpha therapy. Intern Med J.

